# Acute submaximal exercise does not impact aspects of cognition and BDNF in people with spinal cord injury: A pilot study

**DOI:** 10.3389/fresc.2022.983345

**Published:** 2022-11-14

**Authors:** Keegan Nhan, Kendra R. Todd, Garett S. Jackson, Jan W. Van der Scheer, Gabriel U. Dix, Kathleen A. Martin Ginis, Jonathan P. Little, Jeremy J. Walsh

**Affiliations:** ^1^ Brain Exercise Enhancement Laboratory, Department of Kinesiology, McMaster University, Hamilton, ON, Canada; ^2^ School of Health and Exercise Sciences, University of British Columbia, Kelowna, BC, Canada; ^3^ International Collaboration on Repair Discoveries (ICORD), University of British Columbia, Vancouver, BC, Canada; ^4^ The Healthcare Improvement Studies (THIS) Institute, University of Cambridge, Cambridge, United Kingdom; ^5^ Department of Medicine, Division of Physical Medicine and Rehabilitation, University of British Columbia, Vancouver, BC, Canada; ^6^ Centre for Chronic Disease Prevention and Management, University of British Columbia, Kelowna, BC, Canada

**Keywords:** acute exercise, brain-derived neurotrophic factor, SCI exercise guidelines, executive functions, tetraplegia, quadriplegia

## Abstract

**Objective:**

To investigate the effect of acute submaximal exercise, based on the spinal cord injury (SCI) Exercise Guidelines, on cognition and brain-derived neurotrophic factor (BDNF) in people with SCI.

**Design:**

Eight adults (7 males) with traumatic SCI volunteered in this pre-registered pilot study. In randomized order, participants completed submaximal intensity arm cycling (60% of measured peak-power output at 55–60 rpm) for 30 min or time-matched quiet rest (control condition) on separate days. Blood-borne BDNF was measured in serum and plasma at pre-intervention, 0 min and 90 min post-intervention. Cognition was assessed using the Stroop Test and Task-Switching Test on an electronic tablet pre- and 10 min post-intervention.

**Results:**

Submaximal exercise had no effect on plasma [F_(2,12) _= 1.09; *P *= 0.365; *η*² = 0.069] or serum BDNF [F_(2,12) _= 0.507; *P *= 0.614; *η*² = 0.024] at either 0 min or 90 min post-intervention. Similarly, there was no impact of exercise on either Stroop [F_(1,7) _= 2.05; *P *= 0.195; *η*² = 0.065] or Task-Switching performance [F_(1,7) _= 0.016; *P *= 0.903; *η*² < 0.001] compared to the control condition. Interestingly, there was a positive correlation between years since injury and resting levels of both plasma (r = 0.831; *P* = 0.011) and serum BDNF (r = 0.799; *P* = 0.023). However, there was not relationship between years since injury and the BDNF response to exercise.

**Conclusions:**

Acute guideline-based exercise did not increase BDNF or improve aspects of cognition in persons with SCI. This work establishes a foundation for continued investigations of exercise as a therapeutic approach to promoting brain health among persons with SCI.

## Introduction

People with spinal cord injury (SCI) are at an increased risk of experiencing neurocognitive impairment (1). Previous works have observed marked impairments on tests of learning, memory, attention and concentration (2), which may, in part, be due impaired blood pressure regulation negatively impacting cerebral perfusion in SCI (3). Recent estimates suggest that cognitive impairment has a prevalence as high as 60% in people with SCI (1, 4). A wide range of incidence has been reported likely due to heterogeneity in domains of cognition tested (1, 4). What is clear, is that SCI is a chronic condition that may require behavioral approaches to help protect against cognitive impairment (1, 3).

It is well established that exercise training has myriad beneficial effects on cognition and brain function in able-bodied individuals (5). Interestingly, some of the beneficial effects of exercise occur rapidly, as a single bout of moderate intensity aerobic exercise transiently enhances cognitive functions in able-bodied adults including processing speed, inhibitory control, and selective attention (6). Emerging evidence supports that exercise training is also beneficial in people with SCI. Six months of functional-electrical stimulation-assisted exercise significantly improved working memory performance and measures of neurovascular coupling in both people with high- and low-level SCI (7). However, it is currently unknown how a single bout of submaximal aerobic exercise impacts cognition in people with SCI.

A potential mechanism underlying enhanced cognition following acute exercise may, in part, be related to the upregulation of brain-derived neurotrophic factor (BDNF) (8). BDNF plays a key role in neuronal growth, repair and regeneration, and is critical for cognitive functions such as learning and memory (9). Higher levels of circulating BDNF are associated with greater hippocampal volume and memory in humans, which may have implications for protecting against cognitive impairment and neurodegeneration (10). In able-bodied individuals, acute aerobic exercise is a robust stimulus that increases circulating BDNF (11). The evidence in people with SCI is less consistent, with some studies reporting an increase in BDNF (12, 13) and others reporting no change (14–17). The discrepant BDNF responses reported following acute exercise in SCI are likely due to differences in exercise dose (duration and intensity), exercise modality, and participant abilities (injury level/completeness, years since injury) and level of exercise experience (Paralympic athletes vs. inactive individuals). At present, generalizable conclusions regarding the impact of acute exercise on BDNF in people with SCI are not possible and more evidence is needed.

Recently updated exercise guidelines for adults with SCI conditionally recommend 30 min of moderate-to-vigorous aerobic exercise 3 times per week to improve cardiometabolic health and protect against modifiable disease risk (18). The health benefits of these conditional recommendations may also extend to protecting and improving cognitive health in people with SCI; however, the long-term benefits of this exercise dose on cognitive health is unknown, let alone the impact of a single, acute bout of exercise. Accordingly, characterization of the impact of acute exercise is required to establish a foundation for understanding the potential long-term cognitive benefits of guideline-recommended exercise for people with SCI. Therefore, the purpose of this study was to investigate the effect of acute submaximal exercise on cognitive function and circulating BDNF in people with SCI. We hypothesized that acute exercise would increase circulating BDNF levels and enhance aspects of cognition. Findings from this study represent the first characterization of the impact of SCI guideline-recommended exercise on aspects of brain health and provide a foundation for larger trials to further investigate strategies for mitigating the risk of cognitive impairment in an at-risk population.

## Methods

### Participants

Eight individuals with SCI (*n* = 4 with tetraplegia, *n* = 4 with paraplegia) volunteered to participate in a pre-registered trial (clinicaltrials.gov NCT 03955523). Inclusion criteria for this trial were having a ≥1-year post-SCI injury at or below the third cervical vertebrae given that injuries above C3 often indicate insufficient diaphragmatic control and arm functioning to engage in upper-body exercise; able to complete a maximal exercise test based on current American College of Sports Medicine guidelines; self-reported to be meeting the scientific SCI exercise guideline for improving fitness (i.e., 20 min of moderate-to-vigorous intensity aerobic activity two times per week, and strength training two times per week, consisting of three sets of 8–10 repetitions for each major muscle group (18); and have an upper-arm vein capable of accommodating serial blood sampling. Injury completeness was self-reported by participants, based on functional assessment performed by a hospital physician. Loss of sensation and function were confirmed by a researcher during the familiarization visit, following the international standards for neurological classification of SCI established by Kirshblum et al., 2011 (19). Exclusion criteria included: having a clinically diagnosed metabolic (e.g., diabetes) or progressive (e.g., multiple sclerosis) disorder, were pregnant, were training as elite-level athletes, or were unable to refrain from taking anti-inflammatory medication (e.g., ibuprofen) for at least 24 h prior to testing. We did not directly evaluate participants for underlying conditions that may impact cognition or BDNF. Participants completed a general medical screening questionnaire to disclose pre-existing medical conditions and no conditions that may impact cognition or BDNF were reported. The trial conformed with the standards set by the Declaration of Helsinki and subsequent revisions. All participants provided written informed consent prior to commencement of trial activities. This study was approved by the University of British Columbia Clinical Research Ethics Board (H18-03191).

### Peak power output (PPO) aerobic exercise test

Participants completed an aerobic maximal exercise test to determine their PPO using a Lode Angio CPET arm-cycle ergometer (ACE, Groningen, The Netherlands) adjusted for their stature and comfort before experimental trials. During the PPO test, participants were outfitted with a heart rate monitor (Polar, Kempele, Finland) and instructed to maintain a cranking frequency of 55–65 revolutions per minute (rpm) until exhaustion, or for a maximum duration of 30 min. After a 5-min warmup at a self-selected resistance and cadence, the load was increased by 10 Watts (W)/min (paraplegia) or 2 W/min (tetraplegia) starting at 0 W. Ratings of perceived exertion (RPE) were taken during the last 10 s of each minute of exercise using the 6–20 Borg Scale (20), using the adapted script for persons with SCI (21). The test was complete when participants experienced volitional exhaustion or after 30 min (22). After the test, 60% PPO was calculated for every participant and was subsequently used as the work rate for their submaximal exercise bout.

### Experimental protocol

Following the PPO test, participants completed three conditions on separate days in a crossover design with random allocation to the first condition. Conditions were: (1) 2-hour seated control condition; (2) acute exercise condition; and (3) a meal condition. A four-day washout period provided between conditions to ensure no carry-over effects (23). The meal condition data are not a part of the current study as they did not include cognitive function or BDNF analyses and have been reported elsewhere (24). Participants arrived at the lab between 9:00 and 10:00 am following an overnight fast (≥12 h). Following a 10-minute rest period, participants completed cognitive testing on an iPad in a quiet room. Upon completion of cognitive testing, blood was drawn from a vein at the elbow to obtain the baseline measure of BDNF. Participants then completed either the exercise condition or control condition. In the exercise condition, participants completed a 5-minute warmup at a self-selected intensity and were instructed to “warm up the muscles, but not to induce fatigue, breathlessness or exertion”. Following the warm-up, participants completed 30 min of arm-cycling exercise at 60% of their PPO at 55–65 rpm. To characterize perceived exertion, participants reported their RPE during the final 10 s of each minute of exercise. Heart rate was continuously monitored for participant safety during exercise (21) and blood pressure was measured immediately pre- and post- exercise. Upon cessation from exercise, a blood sample was obtained immediately, and participants were given 10 min to recover before completing post-exercise cognitive testing in a quiet room. This period was based on meta-analytical finding showing the greatest effects for post-exercise cognitive enhancement occur when cognition was measured 10–19-min post-exercise (25). Following completion of cognitive testing, participants rested quietly watching a nature documentary until the 90 min post-exercise blood draw. We hypothesized that BDNF would be increased immediately post-exercise compared to rest (11, 26). The 90-min blood draw was taken for metabolic and inflammatory markers reported elsewhere (27). As an exploratory outcome, we assessed BDNF at 90-min to characterize the durability of the hypothesized BDNF response to exercise. Given the dearth of data on BDNF and exercise in SCI, we believe that this preliminary characterization provides important temporal context of the post-exercise period. For the control condition, participants watched a 35-minute nature documentary to match the time spent performing exercise the exercise condition. Otherwise, the timing of blood draws and cognitive testing were identical in the control condition as the exercise condition.

### Cognitive testing protocol

Cognitive function was assessed using the Task-Switching test and the Stroop test. The test battery was administered *via* a tablet-based app (BrainBaseline, Digital Artefacts) during recovery at 10 min post-exercise (and matched timepoints in the seated control condition). The Stroop test involves a word being presented on a screen in various colors. Participants are required to indicate the color of the ink *via* a button press. The Stroop task has 3 different stimuli: (i) congruent – ink color and words are the same (e.g., RED written in red), (ii) incongruent (e.g., RED written in blue) – ink color and words are not the same, and (iii) neutral – word is not related to color (e.g., CAT written in red). The primary endpoint for the Stroop task is Stroop cost (ms) calculated as incongruent trial response time (ms) – congruent trial response time (ms). The Stroop test assesses selective attention and inhibitory control (28). The task-switching test asked participants to switch between two different cognitive tasks depending on the color of the stimulus. For example, if a red box was presented, participants must determine if the number in the box is higher or lower than 5, and if the box was blue, participants must determine if the number was odd or even. The primary endpoint for Task Switch was Switch Cost (ms) calculated as Switch Trial response time (ms) – Stay Trial response time (ms). The Task Switching test assesses cognitive flexibility and an individual's ability to inhibit attentional inertia in the face of changing rules (29).

### BDNF analysis

Both plasma and serum BDNF were measured, given that plasma represents the unbound, bioavailable pool and serum represents unbound and platelet-contained BDNF (30). For serum BDNF, blood was drawn in serum separator tubes and left to clot at room temperature for 1 h (31). Serum tubes were then centrifuged at 1500 g at 4°C for 15 min. For plasma BDNF, blood was drawn in EDTA tubes and centrifuged immediately at 1500 g at 4°C for 15 min. The resultant supernatant was aliquoted and centrifuged at 10,000 g at 4°C for 10 min to yield platelet-poor plasma. Both serum and plasma samples were aliquoted and stored at −80°C until analysis. A commercially available enzyme-linked immunoassay (BEK-2211-2P, Biosensis, SA, Australia) was used to measure BDNF. The intra- and inter-assay coefficients of variation are 1.0% and 5.0%, respectively, as reported by an independent third party (32). Serum BDNF was diluted 100-fold in accordance with the manufacturer's recommendation and confirmed by in-house assay optimization. All other steps were followed in accordance with the manufacturer's instructions.

### Statistical analyses

Data were analyzed by researchers blinded to condition, using JASP (version 0.14.1). Q-Q plots and Shapiro-Wilk tests were used to assess normality and skewness. Data that failed to meet the assumptions of normality were log-transformed. A linear mixed-effects model with fixed effects of condition (exercise vs. control), time (baseline, 0 min post-intervention, 90 min post-intervention), and participants as a random effect was used to compare all BDNF variables. For cognitive function variables, a linear mixed-effects model with fixed effects of condition (exercise vs. control), time (baseline and 10 min post-intervention). Condition order was included as a random effect used to account for potential learning effects due to repeated cognitive testing. Participants were included as a random effect. Effect sizes were calculated as partial-eta square (*η*²) and interpreted based on Cohen's recommendations (small = 0.01; medium = 0.06; large = 0.14) (33). Two-tailed Pearson correlations were run to assess the relationship between BDNF, cognition, and participant characteristics. Statistical significance was set at *P* < 0.05.

## Results

### Participant characteristics

Ten individuals (9 males) with traumatic SCI volunteered to participate in this study. One male participant was unable to complete the experimental protocols due to a pressure sore, unrelated to the trial. A second male participant did not complete the study as the study phlebotomist was unable to collect viable blood samples. The final sample size for the current study was 8 participants (7 males). Participant characteristics are displayed in Table 1.

**Table 1:** Participant characteristics.

Participant	Sex	Age	Time Since Injury (years)	Level of Injury	Motor Complete Injury	Sensory Complete Injury	MAP (mmHg)	Heart Rate (bpm)
1	M	56	11	T7	Yes	Yes	109	83
3	M	32	16	T4-T5	Yes	No	85	76
5	M	26	19	C6/C7	Yes	No	56	80
6	F	56	35	T12-L1	No	No	93	53
7	M	42	25	C5-C6	No	No	61	64
8	M	35	14	C6/C7	Yes	Yes	85	65
9	M	29	8	C6/C7	No	No	85	86
10	M	25	6	T12/L1	Yes	No	99	80

Hemodynamic measures were taken in the resting state. MAP = mean arterial blood pressure.

### Acute submaximal exercise performance

**Figure 1:**
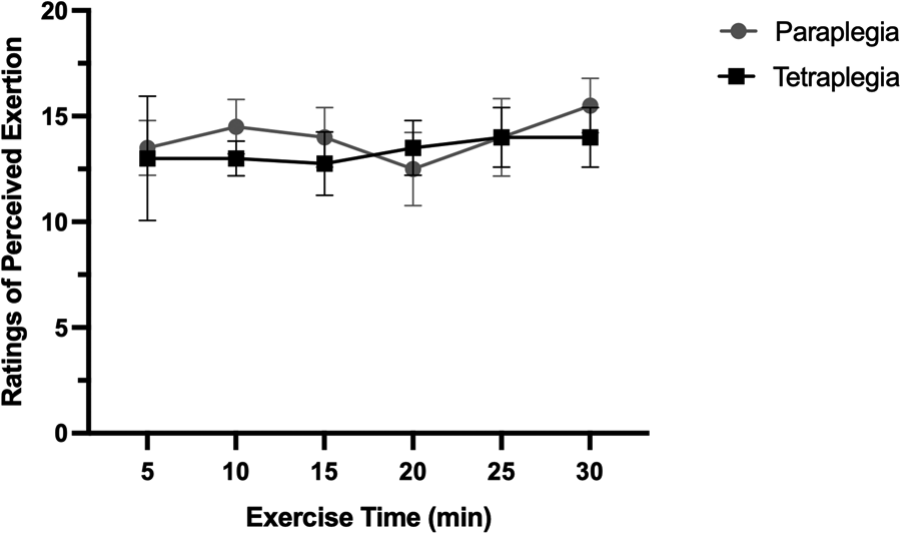
Ratings of perceived exertion over the 30-minute exercise bout in paraplegic and tetraplegic participants.

Exercise intensity for the submaximal exercise bout was set at 60% of an individual's measured PPO. All participants were able to successfully complete the 30-minute bout of submaximal aerobic exercise. Figure 1 displays the average RPE for people with paraplegia and tetraplegia over the 30-minute exercise bout. Overall, there was no change in RPE during the exercise bout and there was no difference in average RPE between groups. Noteworthy, there were differences in the maximum reported RPE (RPE_max_) during exercise, such that people with paraplegia reported significantly higher RPE_max_ (17.25 ± 1.89) compared to people with tetraplegia (14.00 ± 1.4). These data indicate that tetraplegic participants exercised at the targeted submaximal intensity for the entire 30-minute bout. In situations where participants exceeded the submaximal intensity domain, a researcher promptly electronically lowered the work rate to 50% of participants maximum wattage, until their RPE recovered to 12–14, as demonstrated in Figure 1.

### Plasma and serum BDNF

**Figure 2:**
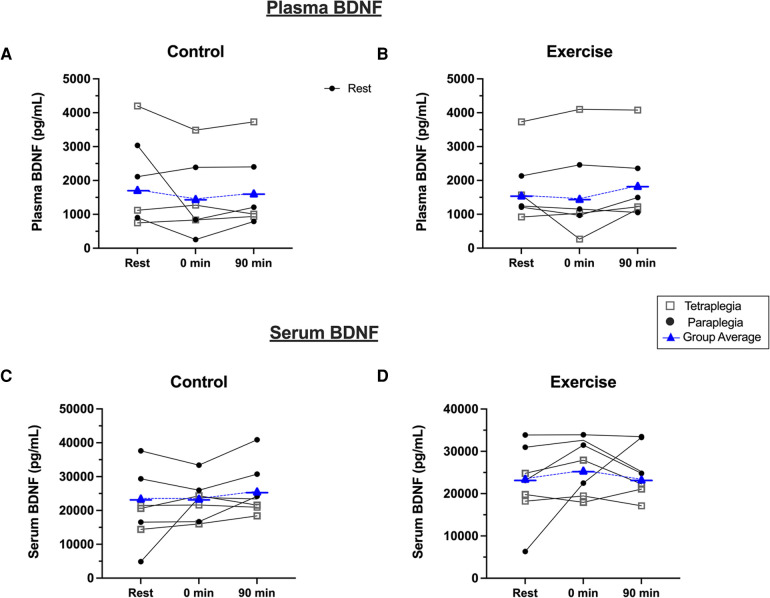
Plasma BDNF response to (**A**) seated control, and (**B**) acute exercise. Serum BDNF response to (**C**) seated control, and (**D**) acute exercise. Black dots are individual responses to each condition. Blue symbols represent group mean responses to each condition.

Due to issues with blood processing, plasma BDNF was assessed in seven participants in the exercise condition and six in the control condition. BDNF data failed tests of normality and were right skewed based on positive Fisher skewness coefficients. Accordingly, we log-transformed plasma BDNF data and subsequently passed normality testing. There was a significant positive relationship between year since injury and basal plasma (r = 0.831; *P* = 0.011) and serum BDNF levels (r = 0.779; *P* = 0.023) (Supplementary Figure S1). Figure 2 shows the response of plasma and serum BDNF from rest to immediately and 90-minutes post-exercise or seated rest. Plasma BDNF did not pass a normality test and data were log-transformed. We observed that there was no main effect of condition [F_(2,12)_ = 0.36; *P* = 0.573; *η*² = 0.009] or time [F_(2,12)_ = 2.37; *P* = 0.135; *η*² = 0.112] on plasma BDNF. Further, there was no time by condition interaction for plasma BDNF [F_(2,12)_ = 1.09; *P* = 0.365; *η*² = 0.069]. Similarly, serum BDNF was not different between conditions [F_(2,12)_ = 0.592; *P* = 0.471; *η*² = 0.024], remained unchanged over time [F_(2,12)_ = 2.25; *P* = 0.148; *η*² = 0.115], and there was not time by condition interaction [F_(2,12)_ = 0.507; *P* = 0.614; *η*² = 0.024].

### Cognitive function

**Figure 3:**
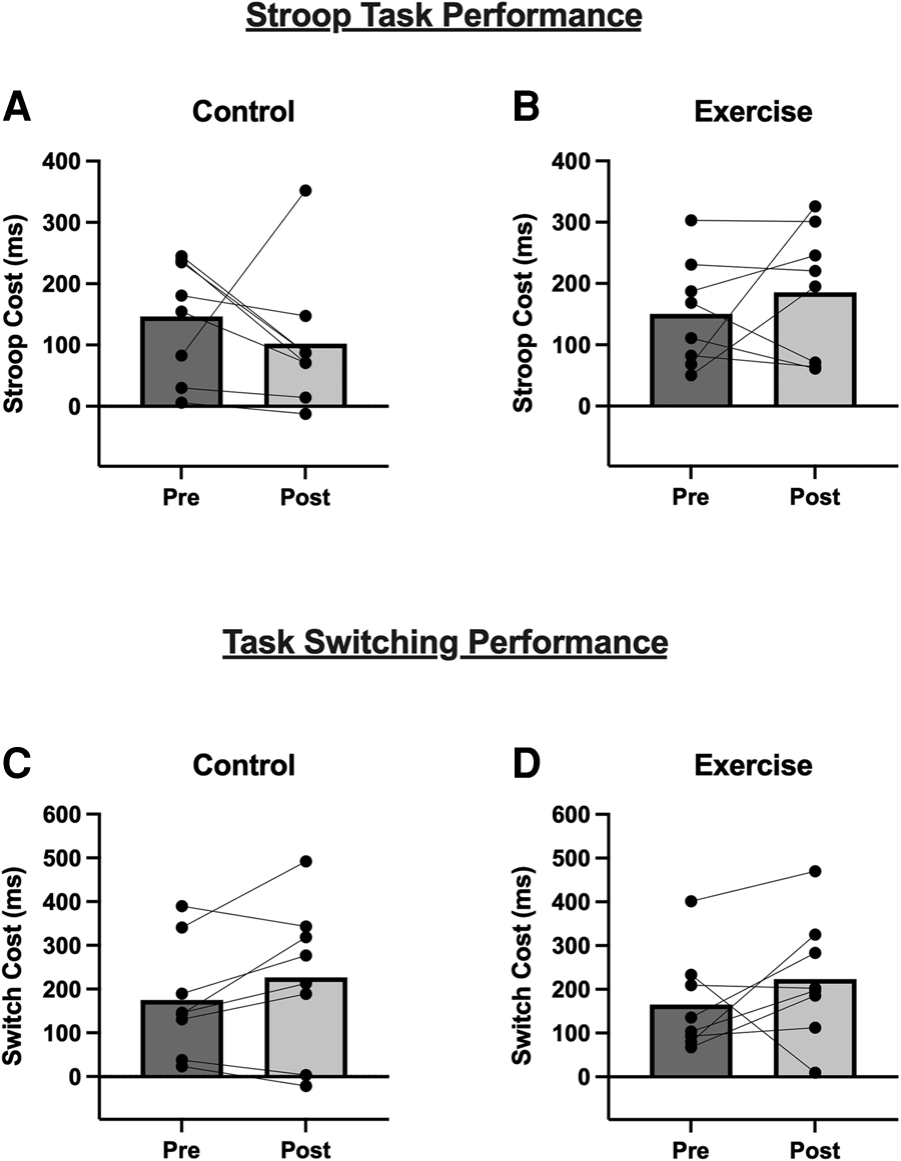
Stroop Task and Task-Switching performance before and 10-minutes after the (**A,C**) Control condition, and (**B,D**) acute exercise. For both outcomes, a lower score (i.e., lower cost) indicates better performance. Bars are group mean data and line-connected dots are individual responses within each condition.

Figure 3 displays performance outcomes for the Stroop Task and Task-Switching Test. After accounting for potential order effects, there was no difference between conditions [F_(1, 7)_ = 1.95; *P* = 0.198; *η*² = 0.077], there was no main effect of time [F_(1, 7)_ = 0.015; *P* = 0.906; *η*² < 0.001], and no time X condition interaction [F_(1, 7)_ = 2.05; *P* = 0.195; *η*² = 0.065] for Stroop performance, as measured by Stroop Cost (ms). Similarly for Task-Switching performance, there was no main effect of condition [F_(1, 7)_ = 0.04; *P* = 0.847; *η*² = 0.002], no main effect of time [F_(1, 7)_ = 2.74; *P* = 0.142; *η*² = 0.135], and no time X condition interaction [F_(1, 7)_ = 0.016; *P* = 0.903; *η*² < 0.001]. Exploratory analysis revealed that there were no significant associations between cognitive performance and BDNF measures at rest or following exercise (Supplementary Table S5).

### Discussion

The purpose of this study was to test the hypothesis that a single bout of submaximal arm cycling exercise would increase BDNF and improve aspects of cognition in people with SCI. To the best of our knowledge, this is the first study to examine the effect of an acute bout of exercise that adheres to the SCI Exercise Guidelines (18) on BDNF and cognition. Contrary to our hypothesis, we found no effect of acute exercise on plasma or serum BDNF during the post-exercise period. Similarly, we observed no effect of acute exercise on cognitive performance as measured by the Stroop Task and Task-Switching Test. Interestingly, we observed a significant positive relationship between BDNF and years since acquiring SCI, suggesting a possible restoration of BDNF levels over time in people with SCI. This study represents an important foundation for future studies to characterize the acute effects of guideline-based exercise on aspects of brain health in people with SCI.

In able-bodied individuals, acute exercise is a robust stimulus that transiently augments circulating BDNF in an exercise intensity- and duration-dependent manner (11). BDNF appears to respond to different exercise modalities, as acute resistance exercise (11) and small muscle mass exercise (34) have been shown to increase BDNF. Changes in BDNF following acute and chronic exercise paradigms have corresponded with improvements in cognition in able-bodied adults (35, 36). Conversely, it remains unclear whether acute exercise has a similar impact on BDNF in people with SCI. Leech and Hornby (2017) observed a significant increase in BDNF following high intensity (100% of peak gait speed) treadmill ramp test (2-min intervals), compared to the moderate and low exercise intensities (66% and 33% of peak gait speed, respectively) in 11 adults with motor incomplete high-level SCI (≥T4). Interestingly, there was a significant positive correlation between RPE and BDNF, which suggests a possible impact of exercise intensity on BDNF in people with SCI. However, there was considerable variability between participants in both gait speed (0.4 to 1.6 m/s) and exercise duration (8–32 min), precluding any conclusions about the impact of exercise dose on BDNF responses. Moreover, exercise was completed on a walking treadmill without body weight assistance, further limiting direct comparison to the current study. Contrastingly, Rojas Vega et al. (13), found that 10 min of low-intensity (54% HR max) handbiking significantly increased serum BDNF compared to rest, whereas BDNF decreased relative to rest following completion of a marathon-distance (42 km) bout of handbiking in 11 elite male athletes with paraplegia (T4-T12). However, the self-paced nature of the marathon “race” condition resulted in a wide range of exercise doses between participants, with an average duration of 85 ± 23 min and exercise intensity of 90% HRmax. Further, responses observed in this trial may not be translatable to a general SCI population as the participants in this study were highly trained athletes with paraplegia. Despite this, evidence from able-bodied individuals somewhat aligns with the findings of Rojas Vega et al. (13). A recent report found that BDNF levels significantly declined 72 h after a marathon race in trained adults, whereas meta-analyses reveal that shorter-bouts (10–40 min) of moderate or high intensity exercise consistently increase BDNF (11).

The present study is the first to investigate the effect of acute submaximal exercise on BDNF using SCI guideline conditional recommendations in people with paraplegia and tetraplegia. Exercise intensity was set at 60% of an individual's PPO for 30 min; however, we observed that people with paraplegia exercised above the submaximal domain and drifted towards near-maximal effort exercise for a short period of time, as indicated by RPE_max_ (23). Intensity was quickly adjusted to return participants to the submaximal domain, and the time spent above submaximal intensity was relatively brief (<2 min). Nonetheless, the discrepancy in relative intensity of exercise between people with paraplegia vs. tetraplegia did not differentially impact the BDNF response (Figure 2), albeit the current trial is underpowered to statistically compare BDNF responses between groups. In able-bodied individuals, BDNF has been shown to increase following a bout of a similar relative intensity of submaximal exercise (11). This may, in part, be facilitated by increased sympathetic nerve activity and vascular shear stress (30). In SCI, autonomic dysfunction and reduced systemic vascular shear stress during acute exercise may impair the BDNF response. We observed a significant positive relationship between years since injury and resting BDNF levels, which we speculate may indicate recovery of BDNF levels over time following post-traumatic SCI. However, there was no relationship between years since injury and the BDNF response to exercise. Future research is needed to systematically characterize the effect of intensity and duration on the acute BDNF response to exercise in SCI. If BDNF cannot be reliably increased with submaximal exercise, other approaches may need to be developed to elicit BDNF to protect brain health in people with SCI.

The beneficial effects of acute exercise in able-bodied individuals on cognition are well described (6). Acute exercise has been shown to enhance multiple cognitive processes such as processing speed, problem-solving, attention, and executive functions in able-bodied individuals (6). Previous work in able-bodied people suggests that an inverted-U relationship exists between exercise intensity and post-exercise cognitive function, with optimal arousal possibly occurring in the moderate intensity domain (37). However, higher physical fitness may buffer the detrimental effects of over-arousal following high-intensity exercise (6). To the best of our knowledge, this was the first study to investigate the effect of acute exercise on cognition in people with SCI. Interestingly, a recent trial provides support for exercise training on cognition in people with SCI (7). Ozturk and colleagues (7) investigated the effect of 6-months of full-body aerobic rowing training on cerebral blood flow and working memory in people with high (≥T4) and low (<T4) level SCI. After 6 months of training, participants with high-level SCI displayed improved reaction time on measures of working memory. Importantly, improvement in reaction time was positively correlated with enhanced cerebral blood flow response during cognitive testing (i.e., neurovascular coupling) and improvements in cardiorespiratory fitness. Accordingly, although we did not observe an immediate effect of acute exercise on cognition, this evidence supports the longer-term benefits of exercise training on cognitive function and brain health in SCI.

Recently, the scientific exercise guidelines for adults with SCI were updated to better reflect the fact that even small doses of exercise can provide health benefits for persons with SCI. As such, the exercise guidelines stipulate adults with SCI should engage in at least 30 min of moderate-to-vigorous aerobic exercise three-times per week to improve cardiometabolic health (18). Given that adults with SCI are at an increased risk of neurocognitive impairment (1), determining whether guideline-based exercise positively impacts cognitive function will add new knowledge pertinent to understanding if guideline-based exercise can be used as an effective and feasible strategy to ameliorate cognitive impairment. This type of research is also important to help inform a wider range of outcomes can be included when formulating future exercise guidelines updates for people with SCI. Indeed, lower levels of BDNF are implicated in cognitive dysfunction in neurodegenerative diseases (38). A recent meta-analysis found that exercise training, regardless of type (aerobic vs. resistance), volume (≥150 min/week vs. <150 min/week), and length (≥12 weeks vs. <12 weeks), significantly increased plasma BDNF levels in people with Parkinson's disease and multiple sclerosis (39). Whether regular exercise training can have a similar long-term impact in people with SCI remains to be determined.

A strength of this study was the inclusion of plasma and serum BDNF as a measure of peripheral BDNF, as inclusion of both plasma and serum BDNF allows for a more complete quantification of the bound and unbound BDNF pool (Walsh and Tschakovsky, 2018). We did not assess *bdnf* genotype in our participants. The Val66Met single-nucleotide polymorphism on the *bdnf* gene impacts activity-dependent release of BDNF (40). Previous research in people with SCI suggests that *bdnf* genotype may contribute to the observed inter-individual variability of the BDNF response to exercise (12). Accordingly, future studies should include measurements of platelets, the amount of BDNF per platelet, and *bdnf* genotype to fully characterize the BDNF response to acute exercise. Limitations of this study include a small sample size, which may have influenced our findings including a greater influence of potential outliers on effect estimates. Although the proportion of males and females in our study was representative of the general population with SCI, greater attempts to characterize potential sex-differences are highly warranted. We assessed cognitive function using the Stroop and Task-Switching Tests, given that executive functions are sensitive to improvement following an acute bout of exercise. Exercise work rate was set at 60% of an individual's PPO; however, paraplegic participants exercised at relatively higher intensity for short periods of the exercise bout, which may have contributed variability to the observed post-exercise cognitive responses. It is possible that other cognitive domains are positively impacted by acute exercise in people with SCI and warrants follow-up with a more comprehensive cognitive testing battery, if feasible. Further, participants were tested in the fasted state to reduce variability in BDNF measures; however, this may have impacted their cognitive responses to exercise.

In conclusion, our study is the first to investigate the scientific SCI exercise guidelines on BDNF and cognition in adults with SCI. This line of research is imperative as guideline-based has been shown to improve cardiometabolic health and physical fitness; however, the impact on brain health in people with SCI is unknown. We found that an acute bout of guideline-based exercise did not increase BDNF and did not improve aspects of cognitive function in adults with SCI. Results from this study provide guidance for future researchers to comprehensively evaluate the impact of following the SCI exercise guidelines on brain health among persons with SCI. This has significant implications for protecting and improving brain health across the lifespan in people with SCI.

## Data Availability

The original contributions presented in the study are included in the article/Supplementary Material, further inquiries can be directed to the corresponding author/s.
