# Supplementary material for: Acute submaximal exercise does not impact aspects of cognition and BDNF in people with spinal cord injury: A pilot study
Source: Front Rehabil Sci. 2022 Nov 14;3:983345. doi: 10.3389/fresc.2022.983345 (PMC9701830; doi:10.3389/fresc.2022.983345)
Supplement: Supplementary file 1 [file Datasheet1.docx]

**Supplementary Figures and Tables**


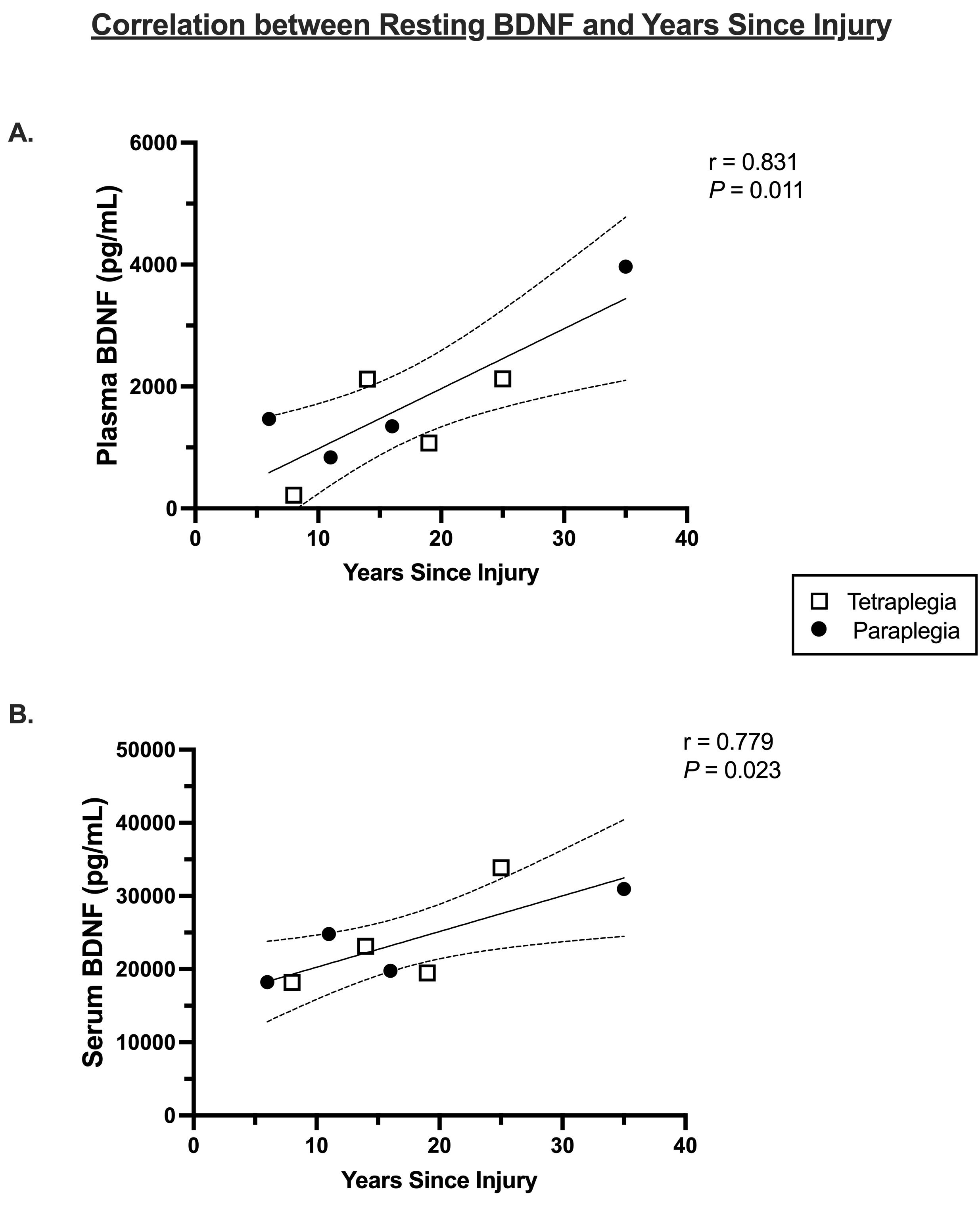


**Supplementary Figure 1.** Correlation between years since injury and resting plasma (A) and serum (B) BDNF in people with tetraplegia (n = 4; open squares) and paraplegia (n = 4; closed circles).

**Supplementary Table 1.** BDNF Responses in Adults with Paraplegia

|  | Rest | 0 min | 90 min |
| --- | --- | --- | --- |
| **Plasma BDNF (pg/mL)** | | | |
| Control Condition | 1556.6 (1270.0) | 1161.9 (1101.5) | 1469.2 (836.7) |
| Exercise Condition | 1214.1 (763.6) | 1193.4 (943.0) | 1636.1 (662.8) |
| **Serum BDNF (pg/mL)** | | | |
| Control Condition | 26461.9 (9089.3) | 23926.6 (7409.8) | 29224.2 (8738.6) |
| Exercise Condition | 23671.9 (7113.3) | 26477.8 (7345.8) | 25400.9 (5810.2) |

Data are mean (SD).

**Supplementary Table 2.** BDNF Responses in Adults with Tetraplegia

|  | Rest | 0 min | 90 min |
| --- | --- | --- | --- |
| **Plasma BDNF (pg/mL)** | | | |
| Control Condition | 1897.2 (1567.2) | 1681.7 (1216.4) | 1737.9 (1336.4) |
| Exercise Condition | 1912.0 (1243.9) | 1733.2 (1661.4) | 2000.7 (1396.7) |
| **Serum BDNF (pg/mL)** | | | |
| Control Condition | 20668.1 (4844.9) | 23179.3 (6123.1) | 22088.2 (3805.9) |
| Exercise Condition | 23448.4 (5746.6) | 24488.0 (6981.9) | 21316.9 (3335.0) |

Data are mean (SD).

**Supplementary Table 3.** Cognitive Performance in Adults with Paraplegia

|  | Rest | Post-Intervention |
| --- | --- | --- |
| **Task Switching Performance (Switch Cost; ms)** | | |
| Control Condition | 121.6 (70.3) | 190.7 (151.5) |
| Exercise Condition | 96.7 (29.9) | 247.9 (67.58) |
| **Stroop Performance (Stroop Cost; ms)** | | |
| Control Condition | 119.5 (97.9) | 124.6 (157.9) |
| Exercise Condition | 99.6 (52.6) | 163.3 (124.4) |

Data are mean (SD).

**Supplementary Table 4.** Cognitive Performance in Adults with Tetraplegia

|  | Rest | Post-Intervention |
| --- | --- | --- |
| **Task Switching Performance (Switch Cost; ms)** | | |
| Control Condition | 173.5 (100.0) | 80.0 (54.9) |
| Exercise Condition | 200.9 (92.3) | 207.7 (101.6) |
| **Stroop Performance (Stroop Cost; ms)** | | |
| Control Condition | 228.6 (165.1) | 263.1 (207.2) |
| Exercise Condition | 243.2 (127.2) | 198.6 (197.3) |

Data are mean (SD).

**Supplementary Table 5.** Exploratory Correlations between Cognitive Performance and BDNF

| **Control Condition** | **Rest** | **Post-Intervention** |
| --- | --- | --- |
| pBDNF vs. Stroop Cost | r = -0.260; *P* = 0.534 | r = 0.625; *P* = 0.134 |
| sBDNF vs. Stroop Cost | r = -0.634; *P* = 0.091 | r = 0.037; *P* = 0.931 |
| pBDNF vs. Switch Cost | r = -0.202; *P* = 0.631 | r = -0.569; *P* = 0.182 |
| sBDNF vs. Switch Cost | r = -0.349; *P* = 0.397 | r = -0.493; *P* = 0.214 |
| **Exercise Condition** | **Rest** | **Post-Intervention** |
| pBDNF vs. Stroop Cost | r = 0.360; *P* = 0.381 | r = 0.200; *P* = 0.635 |
| sBDNF vs. Stroop Cost | r = 0.140; *P* = 0.742 | r = -0.377; *P* = 0.357 |
| pBDNF vs. Switch Cost | r = -0.099; *P* = 0.815 | r = -0.332; *P* = 0.421 |
| sBDNF vs. Switch Cost | r = -0.400; *P* = 0.326 | r = -0.494; *P* = 0.214 |

pBDNF = plasma BDNF; sBDNF = serum BDNF
